# Association of Vitamins D, B_9_ and B_12_ with Obesity-Related Diseases and Oral Microbiota Composition in Obese Women in Croatia

**DOI:** 10.17113/ftb.60.02.22.7478

**Published:** 2022-06

**Authors:** Ana Huđek Turković, Martina Matovinović, Kristina Žuna, Lucija Škara, Snježana Kazazić, Višnja Bačun-Družina, Ksenija Durgo

**Affiliations:** 1 Faculty of Food Technology and Biotechnology University of Zagreb, Pierottijeva 6, 10000 Zagreb, Croatia; 2 Department of Internal Medicine, Division of Endocrinology, Croatian Obesity Treatment Referral Centre, University Hospital Centre Zagreb, Kišpatićeva 12, 10000 Zagreb, Croatia; 3 School of Medicine University of Zagreb, Šalata 3, 10000 Zagreb, Croatia; 4 Ruđer Bošković Institute, Bijenička cesta 54, 10000 Zagreb, Croatia

**Keywords:** obesity-related diseases, oral microbiota composition, vitamin D, vitamin B_12_, folic acid

## Abstract

**Research background:**

Oral microbiota has become an important factor in obesity, but its association with obesity-related diseases and serum 25-hydroxy vitamin D [25(OH)D] and B complex amounts is still uncertain. The main aim of the paper is to determine the variation in oral microbiota composition as a response to the vitamin status and obesity-related diseases in obese females from Croatia. We hypothesized that the prevalence of probiotic or pathogenic bacteria in the oral cavity of obese women in Croatia depends on the amounts of vitamin B_9_ (folic acid), B_12_ and 25(OH)D in serum and/or hypertension, diabetes and prediabetes diagnosis.

**Experimental approach:**

To test the defined research hypothesis, female individuals with body mass index (BMI)≥30 kg/m^2^ (*N*=70) were recruited to participate in this study. Obese women were divided into groups according to BMI value, diagnosis of obesity-related diseases and amount of micronutrient in blood. For the quantitative determination of folic acid, vitamin B_12_ and 25(OH)D in serum, an electrochemiluminescence protein binding assay (ECLIA) was performed. Microorganisms isolated from the saliva of obese women were analyzed by MALDI-TOF mass spectrometer.

**Results and conclusions:**

The presented results do not support the hypothesis that the prevalence of probiotic or pathogenic bacteria in the oral cavity of obese women in Croatia depends on the amount of micronutrients. On the other hand, hypertension and diabetes/prediabetes favour the growth of oral pathogens, specifically increased levels of *Candida* sp.

**Novelty and scientific contribution:**

To the best of our knowledge, this is the first study showing the relationship between obesity, micronutrient amount, oral microbiota composition, and the incidence of obesity-related disease. We included only obese women from Croatia, so it is regionally specific. Also, we have shown that oral microbiota composition is not connected with micronutrient deficiencies but only with obesity-related diseases.

## INTRODUCTION

Obesity is a complex disease and a great medical problem that increases the risk of developing comorbidities such as heart disease, prediabetes, diabetes, high blood pressure and cancer (*1*). The association between diabetes and obesity is clearly visible from the fact that as many as 80% of people with type 2 diabetes are obese and, from the opposite perspective, that 10% of obese people have type 2 diabetes (*2*). However, the mechanisms underlying obesity associated with diabetes and prediabetes or other obesity-related diseases like hypertension have not yet been defined. The most common strategies for preventing prediabetes, diabetes and hypertension include lifestyle modifications and pharmacological interventions. Still, there are indices that the development of the diseases is in tight connection with a deficit of certain micronutrients, including D and B complex vitamins. Fat-soluble 25-hydroxy vitamin D has anti-inflammatory and immunomodulatory properties, and it can reduce insulin resistance (*3*). More specifically, it increases the sensitivity of fat and muscle tissue to existing insulin and decreases gluconeogenesis in the liver, which is an additional contribution to reducing blood sugar concentrations (*4*).

Probiotic bacteria, mainly belonging to the genera *Lactobacillus* and *Bifidobacterium*, besides many health benefits, can produce vitamins (*5*) such as folate (*6*), and some other members of the gut microflora can produce cobalamin (B_12_). Folic acid and vitamin B_12_ are both required for normal cell division because of their involvement in DNA synthesis and amino acid metabolism. Additionally, folic acid is involved in DNA repair through *de novo* DNA synthesis and methylation, while vitamin B_12_ deficiency leads to fatty acid metabolism dysregulation (*7*).

New findings suggest a causal link between microbial dysbiosis (which is responsible for the chronic systemic inflammation that affects insulin resistance) and obesity, indicating the change in the intestinal microbiota composition in obese subjects, with an increase in the phylum *Firmicutes* and a decrease in the phylum *Bacteroidetes*, which is associated with the high-fat diet and dysbiosis of gut microbiota (*8*).

Oral microbes play an important role in maintaining systemic and oral health by resistance to microbial colonization, digestion of nutrients and immune response (*9*). Based on bacterium-mediated inflammatory processes, changes in the oral microbiome appear to be connected with obesity. It has been observed that with an increase in BMI, the diversity in bacterial species in saliva decreases (*10*-*12*).

In this work, the changing trend of oral microbiota composition depending on serum 25(OH)D, vitamin B_12_ and folic acid amounts or presence of diabetes/prediabetes and hypertension in obese women in Croatia is investigated. Furthermore, we defined the association among probiotic or pathogen oral microbiota, obesity and obesity-related diseases.

## MATERIALS AND METHODS

This study was conducted over a period of one year (2020-2021) by the Faculty of Food Technology and Biotechnology, University of Zagreb, Croatia, and University Hospital Centre Zagreb, Zagreb, Croatia. All scientific procedures were approved by the Ethics Committee of the University Hospital Centre Zagreb, Croatia (Permit class: 8.1-18/161-2, No. 02/21 AG). In accordance with ethical principles, all of the patients signed an informed consent form before undergoing saliva and blood sampling procedures.

### Subjects and study design

**Fig. 1:**
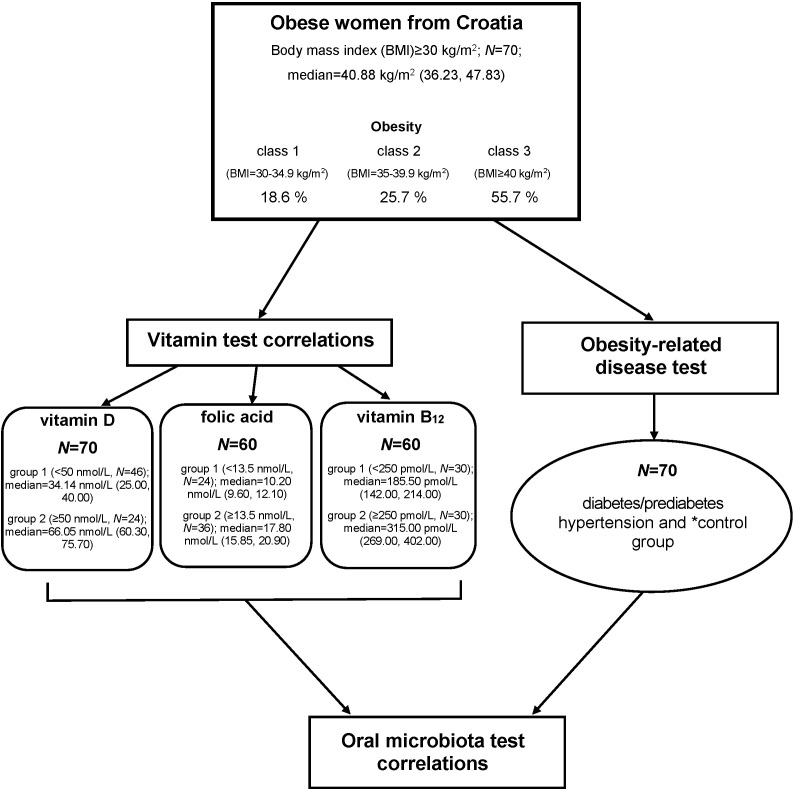
Flowchart of participant selection for the analysis. *Control group represents a female population without diabetes/prediabetes and/or hypertension

The experimental population consisted of obese women from Croatia aged 20 to 74 (*N*=70, median (age=45 (37, 55; 25th and 75th percentile). All participants were patients of the Croatian Obesity Treatment Referral Centre of the Division of Endocrinology, University Hospital Centre Zagreb. We focused only on obese women with BMI≥30 kg/m^2^, who were subdivided into three classes according to BMI range (*13*). Association of each BMI class with hypertension classification, diabetes/prediabetes diagnosis and the amount of micronutrients in blood were observed. Fig. 1 shows the study design. In *a priori* power analysis, *t*-test was used and minimum sample size using G*Power v. 3.1.9.7 software (Heinrich Heine University, Duesseldorf, Germany; http://www.gpower.hhu.de/) was calculated (*14*). Accordingly, the minimum sample size with 0.80 prespecified effect size (d) to detect a difference of 0.8 standard deviation, 0.05 significance level (α) and 0.80 power level (1-β) was calculated to be 21 per group. Considering that, we decided to include 70 voluntary participants in this study as an appropriate sample size to have a possible significant difference between the two or three groups at the end, depending on the examined parameter.

This study included only females who were not taking any forms of supplements that contained vitamin D, B_12_ or folic acid. Furthermore, exclusion criteria were pituitary and/or adrenal disease, untreated thyroid disease, prior myocardial infarction, stroke, transient ischemic attack or oncological disease. All analyses were performed during the first visit of obese patients to the Croatian Obesity Treatment Referral Centre (University Hospital Centre Zagreb, Croatia) without prior education in proper and balanced nutrition and controlled diet. Therefore, the diet of the patients was without accurate data on the composition and daily intake of calories. We included only female patients for several reasons: the Croatian Obesity Treatment Referral Centre has a much higher prevalence of obese female than male patients; gut microbiota differences between men and women, which can be influenced by the grade of obesity (*15*); the observed divergence could play a dominant role in defining gender differences in the incidence of metabolic and intestinal inflammatory diseases; studies in humans have produced conflicting results due to high inter-individual heterogeneity in age, diet and hormonal factors, and the largely unexplored influence of gender (*16*). Total body mass (kg), fat (%), fat mass (kg), muscle (%) and BMI (kg/m^2^) of the study participants were measured with the Tanita body fat scale (Tanita, Tokyo, Japan) using bioelectrical impedance analysis at the University Hospital Centre Zagreb.

### Vitamin- and obesity-related test correlations

The 25(OH)D values in serum below 50 nmol/L represented a deficiency, between 50.1 and 75 nmol/L were defined as an insufficiency, and anything above 75 nmol/L was considered as a sufficient level of serum 25(OH)D in a human adult (*17*). Folic acid values between 13.5 and 45.3 nmol/L are considered normal range and above 45.3 nmol/L are elevated folic acid concentrations. When folic acid concentrations in the blood are less than 6.8 nmol/L, it is considered a folic acid deficiency. Insufficient folic acid concentrations are between 6.8 and 13.4 nmol/L (*18*). It has been well-established that people with vitamin B_12_ concentrations in blood between 200 and 350 pg/mL (147.6–258.3 pmol/L) have clear vitamin deficiency symptoms (*19*). Thus, in this study a cut-off value was 250 pmol/L. Fig. 1 shows the grouping of obese women according to vitamin concentrations.

Classification of obese women according to obesity-related disease was made using the following criteria: only diabetes/prediabetes diagnosis, only hypertension diagnosis, and without diseases (control group; Fig. 1).

### Saliva and blood sampling

For analytical procedures, saliva and blood samples of obese female patients were taken at the Division of Endocrinology, University Hospital Centre Zagreb, Croatia. Venous blood sampling (5 mL) was performed in VACUETTE^®^ tube CAT Serum Separator Clot Activator (Greiner Bio-One GmbH, Kremsmünster, Austria). The 25-hydroxy vitamin D, B_12_ and folic acid concentrations were determined in the serum of fresh pooled blood samples. Furthermore, after rinsing the mouth with 15 mL of sterile saline solution to prevent the effect of previously eaten food on the microorganisms in the oral cavity, 2 mL of saliva samples were collected using sterilized centrifuge tubes (TPP, Trasadingen, Switzerland) and stored at −20 °C (*20*).

### Isolation and identification of oral microorganisms

The isolation and identification procedures were previously described by Huđek *et al.* (*20*). Original saliva and a 1:1 dilution of saliva (0.5 mL) in phosphate buffer (pH=7.2) were inoculated on nutrient agar plates (de Man-Rogosa-Sharpe and Luria-Bertani). Furthermore, samples were incubated for two days under aerobic conditions in a thermostat at 37 °C. Microflex LT matrix-assisted laser desorption ionization time-of-flight (MALDI-TOF) mass spectrometer (Bruker Daltonik, Bremen, Germany) was used for the identification of colonies with different phenotypes. MALDI Biotyper 3.0 software package (Bruker Daltonik) with standard settings was used to process the recorded mass spectra.

In this work we isolated 118 different species of bacteria (5 different phyla: *Firmicutes, Actinobacteria, Proteobacteria, Bacteroidetes* and *Fusobacteria*) and 13 different species of fungi (2 different phyla: *Ascomycota* and *Basidiomycota*), 34 potentially pathogenic and 22 potentially probiotic bacteria (Table 1). The classification of probiotic bacteria was adapted according to Fijan (*21*) and pathogens according to Werth (*22*). For the statistical analyses, ’microbiota function’ presents the number of different species that fit into each of three categories – probiotic, pathogen or none. Bacteria were identified as binary variables (present/not present).

**Table 1:** Oral bacteria and fungi isolated from the saliva of obese women from Croatia. The classification of probiotic bacteria was made according to Fijan (*21*; marked in green) and pathogens according to Werth (*22;* marked in red)

**Bacteria**					**Fungi**
***Firmicutes***			***Proteobacteria***	***Actinobacteria***	***Ascomycota***
*Streptococcus salivarius*	*Clostridium cochlearium*	*Lactobacillus rhamnosus*	*Pseudomonas abietaniphila*	*Rothia aeria*	*Candida albicans*
*Streptococcus oralis*	*Bacillus cereus*	*Lactobacillus fermentum*	*Pseudomonas anguilliseptica*	*Rothia amarae*	*Candida kyfer*
*Streptococcus parasanguinis*	*Bacillus infantis*	*Lactococcus lactis*	*Pseudomonas brenneri*	*Rothia dentocariosa*	*Candida krusei*
*Streptococcus mitis*	*Bacillus pumilus*	*Lactobacillus paracasei*	*Pseudomonas fluorescens*	*Rothia mucilaginosa*	*Candida glabrata*
*Streptococcus cristatus*	*Bacillus licheniformis*	*Lactobacillus plantarum*	*Pseudomonas oryzihabitan*	*Kocuria kristinae*	*Candida tropicalis*
*Streptococcus pyogenes*	*Aerococcus viridians*	*Lactobacillus sharpeae*	*Pseudomonas thivervalensis*	*Kocuria palustris*	*Candida dubliniensis*
*Streptococcus didelphis*	*Brochothrix thermosphacta*	*Lactobacillus sakei*	*Pseudomonas veronii*	*Micrococcus luteus*	*Candida inconspicua*
*Streptococcus vestibular*	*Gemella sanguinis*	*Lactobacillus curvatus*	*Burkholderia anthina*	*Brevibacterium celere*	*Candida parapsilosis*
*Streptococcus dysgalactiae*	*Kandleria vitulina*	*Lactobacillus intestinalis*	*Burkholderia fungorum*	*Corynebacterium durum*	*Clavispora lusitaniae*
*Streptococcus ferus*	*Leuconostoc pseudomesenteroides*	*Lactobacillus nagelii*	*Burkholderia thailandensis*	*Microbacterium paraoxydans*	*Kluyveromyces marxianus*
*Streptococcus orisratti*	*Pediococcus pentosaceus*	*Lactobacillus zeae*	*Burkholderia tuberum*	*Microbacterium saperdae*	*Lodderomyces elongisporus*
*Streptococcus ratti*	*Veillonella atypica*	*Lactobacillus casei*	*Acinetobacter gerneri*	*Jonesia denitrificans*	*Penicillium camemberti*
*Streptococcus phocae*	*Staphylococcus aureus*	*Lactobacillus salivarius*	*Acinetobacter lwoffii*	*Propionibacterium avidum*	***Basidiomycota***
*Streptococcus australis*	*Staphylococcus epidermidis*	*Lactobacillus ruminis*	*Acinetobacter pittii*	***Bacteroidetes***	*Cryptococcus magnus*
*Streptococcus canis*	*Staphylococcus hominis*	*Lactobacillus crispatus*	*Acinetobacter radioresistens*	*Chryseobacterium scophthalmum*	
*Streptococcus equinus*	*Staphylococcus warneri*	*Lactobacillus parabuchneri*	*Neisseria flavescens*	***Fusobacteria***	
*Streptococcus marimammalium*	*Staphylococcus pasteuri*	*Lactobacillus murinus*	*Neisseria mucosa*	*Fusobacterium naviforme*	
*Streptococcus infantis*	*Staphylococcus saprophyticus*	*Lactobacillus kalixensis*	*Neisseria perflava*		
*Streptococcus sanguinis*	*Staphylococcus haemolyticus*	*Lactobacillus delbrueckii*	*Escherichia coli*		
*Streptococcus gordonii*	*Staphylococcus xylosus*	*Enterococcus dispar*	*Herbaspirillum huttiense*		
*Streptococcus lutetiensis*	*Staphylococcus equorum*	*Enterococcus faecalis*	*Moraxella catarrhalis*		
*Streptococcus porcinus*	*Staphylococcus saccharolyticus*	*Clostridium cochlearium*	*Rhizobium radiobacter*		
*Streptococcus anginosus*	*Staphylococcus capitis*		*Rhizobium rubi*		
*Streptococcus criceti*	*Staphylococcus simulans*		*Alcaligenes faecalis*		
*Streptococcus pneumoniae*			*Granulicatella adiacens*		
*Streptococcus intermedius*			*Leminorella richardii*		
*Streptococcus gallinaceus*			*Ochrobactrum grignonense*		
			*Pandoraea pulmonicola*		
			*Pluralibacter pyrinus*		
			*Serratia marcescens*		
			*Shewanella baltica*		

### Laboratory blood testing

The laboratory where all analyses were performed has HAA accreditation (Croatian Accreditation Agency). The Roche Diagnostics total vitamin D assay as competitive electrochemiluminescence protein binding assay (ECLIA) was used for the quantitative determination of 25(OH)D, vitamin B_12_ and folic acid in human serum samples (*23*, *24*). Further, the ECLIA assay was used for the *in vitro* quantitative determination of vitamins on a Cobas 6000cee analyzer (Roche Diagnostics, Mannheim, Germany). The method used for serum 25(OH)D measurement was verified with an assay validated by the Vitamin D External Quality Assessment Scheme (DEQAS; http://www.deqas.org/) (*25*). The validation of methods used for folic acid and vitamin B_12_ measurement was performed by DGKL (The German Society for Clinical Chemistry and Laboratory Medicine e.V.) at the Reference Institute for Bioanalytics in Bonn.

#### Data analysis and statistics

First, the one-sample Kolmogorov-Smirnov normality test was used to determine if a data set is normally distributed. Correlations among variables including BMI, fat and muscle mass fractions, fat mass, age, 25(OH)D, folic acid, vitamin B_12_, microbiota classification (probiotic, none and pathogen), hypertension classification and prediabetes/diabetes diagnosis were determined using correlation matrix and Spearman’s test. We used factorial ANOVA to determine statistically significant interactions between independent variables of the oral microbiota composition and diabetes-related diseases or vitamin concentrations. Multiple comparisons were adjusted using Tukey’s honestly significant difference (HSD) test. Statistically significant values were those that differed at p<0.05. Statistical analysis was carried out using IBM SPSS v. 22 software (*26*).

## RESULTS AND DISCUSSION

In this study, only the female population was involved because obesity has a much higher prevalence in female than male patients. Also, results obtained in female and male population cannot be compared because there are significant gut microbiota and hormonal status differences, which impact metabolic and intestinal inflammatory disease development.

### Correlations among obesity factors, vitamin concentration in blood and obesity-related diseases

In this study, BMI (kg/m^2^) values positively correlated with a fat mass fraction (%), fat mass (kg) and age, while a negative correlation was demonstrated with serum 25(OH)D concentrations and muscle mass fractions (%) (Table 2 (*27*)). The same correlations among BMI, fat mass fraction (%), fat mass and muscle mass fraction (%) have been confirmed in other worldwide studies with obese individuals (*28*, *29*). A statistically significant positive correlation was found between serum 25(OH)D and folic acid concentrations in the tested obese women (Table 2). According to a recent meta-analysis, the prevalence of vitamin D deficiency is 35% higher in people with obesity than in people with normal body mass (*30*). Through a complex chemical process, it is possible that excess body fat requires a surplus of vitamin D, which diminishes the amount available for other processes. Gominak (*31*) showed that vitamin D deficiency altered the intestinal microbiome, reducing vitamin B production in the gut. As well as for vitamin D, Kerns *et al.* (*32*) have confirmed that obese individuals have a deficiency in vitamin B_9_ at a very high percentage (16-29%). Obese women in Croatia follow the worldwide trend of a correlation between higher obesity and lower 25(OH)D concentrations in blood (*33*).

**Table 2:** Descriptive statistics and Spearman’s rank correlation matrix for selected variables

		Mean	SD	1	2	3	4	5	6	7	8	9	10	11
1	Age	46.10	12.20											
2	Body mass index	42.20	8.09	0.408**										
3	*c*(25(OH)D)/(nmol/L)	44.78	20.02	-0.048	-0.273*									
4	*c*(folic acid)/(nmol/L)	15.63	5.86	0.021	-0.135	0.293*								
5	*c*(vitamin B_12_)/(pmol/L)	270.53	128.31	0.020	-0.221	0.198	0.248							
6	Hypertension classification	0.53	0.50	0.514**	0.402**	0.127	-0.027	0.026						
7	Diabetes/prediabetes diagnosis	0.44	0.50	0.225	0.251*	-0.023	-0.027	0.019	0.208					
8	Microbiota function	0.84	0.67	0.159	0.196	0.085	0.059	0.025	0.319**	0.684**				
9	*w*(fat)/%^#^	46.51	6.11	0.082	0.488*	0.073	0.106	0.143	0.196	0.142	0.095			
10	*m*(fat)/kg	55.01	16.04	0.254	0.776**	0.086	0.097	0.130	0.290	0.130	0.011	0.705**		
11	*w*(muscle)/%	48.64	7.47	-0.115	-0.685**	-0.175	-0.034	-0.006	-0.048	0.039	0.036	-0.698**	-0.573**	

*N*=70 (except for folic acid and vitamin B_12_ *N*=60). Hypertension classification: 0=none, 1=hypertension; Diabetes/prediabetes diagnosis: 0=none, 1=diabetes/prediabetes; Microbiota function (the number of different species that fit into each of three defined categories; for example, probiotic is represented by the variable of how many probiotic species were detected on the swab of the oral cavity): 0=probiotic, 1=none, 2=pathogen. *p<0.05, **p<0.01. *^#^*At the same body mass index (BMI), women usually contain ~10% more body fat than men (*27*)

**Fig. 2:**
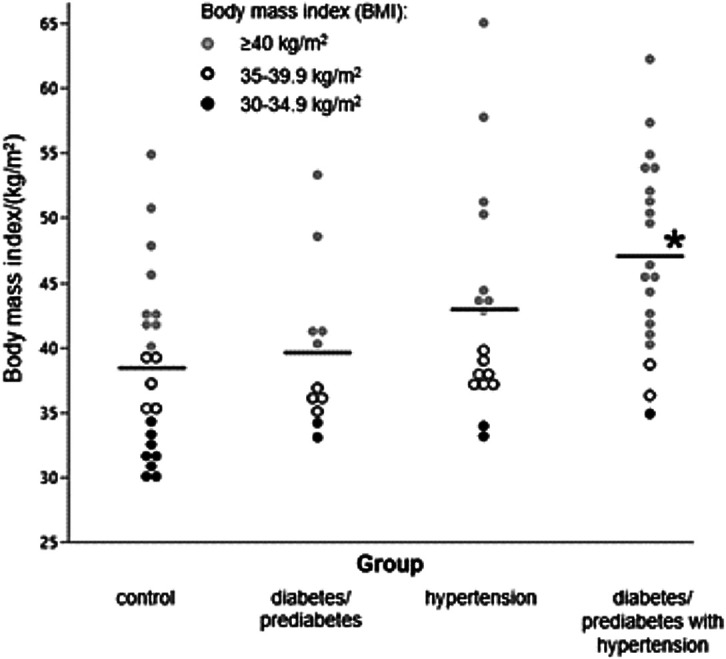
Scatter plot visualizing the relationship between the control group (no obesity-related disease diagnosis) and obese women from Croatia with diabetes/prediabetes, hypertension or both diagnoses based on body mass index (BMI) values. The black line represents the median value in each group while the black circles symbolise class 1 (BMI=30-34.9 kg/m^2^), white circles class 2 (BMI=35-39.9 kg/m^2^) and grey circles class 3 obesity (BMI≥40 kg/m^2^). *Statistically different values compared to the control group (p<0.05)

In this study, we showed a statistically significant positive correlation between the BMI values and incidence of hypertension and diabetes/prediabetes diagnosis (Table 2). By dividing the tested obese women into 3 categories according to obesity-related diseases, a statistically significant difference in BMI values was determined between the obese women without any disease and the group of women with both hypertension and prediabetes/diabetes diagnosis (p<0.01; Fig. 2). As in our study, results from two national surveys have shown that the prevalence of hypertension and diabetes occurred across all ranges of BMI and increased with higher BMI (*34*). In vitamin-D-deficient patients with a diabetes diagnosis, supplementation of vitamin D increased serum 25-(OH)D, sirtuin 1, irisin and glycosylated haemoglobin concentrations, and these improvements may reduce insulin resistance. Furthermore, vitamin D can increase transport of glucose in the gut and insulin receptor gene expression in β-cells, while intestinal calcium absorption may serve as a stimulus for insulin release. Vitamin D deficiency, obesity and oxidative DNA damage are significant predictors of genomic instability (*34*). Vitamin D can control gene expression *via* genomic and epigenomic mechanisms, and that could be a reason why it has such wide-ranging non-skeletal health benefits (*35*). Epigenetic effect of 25(OH)D is linked to histone modifications, mainly acetylation. However, there is a study that showed that serum 25(OH)D could change the methylation ratio of the *CYP2R1* gene, which is a potential candidate for predicting serum 25(OH)D variation (*36*).

### Relationship of oral microbiota composition and concentration of vitamin in blood with obesity or obesity-related diseases

There was a positive correlation between the number of oral *Proteobacteria* and *Firmicutes* bacteria (Spearman’s rho CC=0.284, p=0.017). Zeigler *et al.* (*12*) also reported bacteria from *Proteobacteria* phylum and individual *Neisseria mucosa* to be present in sixfold higher amounts in the saliva of obese adolescent subjects. Furthermore, Yang *et al.* (*37*) showed that *Proteobacteria* and *Firmicutes* bacteria were significantly associated with an increased obesity prevalence in obese individuals from the Southern Community Cohort Study, USA.

What should be emphasized first is that there is a positive correlation between the incidence of hypertension or prediabetes/diabetes and pathogenic bacteria in the oral cavity in obese women from Croatia (Table 2). In obese women with diabetes/prediabetes, there is a greater variety of fungi (number of different species) of the genus *Candida* than all 13 isolated fungi in this study (Mann-Whitney *U*=462.00, p=0.04). The identification results of the oral microbiota showed the predominance of *Candida* sp. in the *Ascomycota* phylum. The higher BMI values (if we compare only class 1 and class 3 obesity levels), depending on the presence of diabetes/prediabetes, positively affected the number of *Ascomycota* in the oral cavity (factorial ANOVA, *F*=4.091, p=0.021). Furthermore, a statistically significant difference in the number of *Ascomycota* among different obesity levels was seen only in the diabetes/prediabetes group (Tukey’s HSD *post hoc* multiple comparison for diabetes/prediabetes group: comparison between class 1 and class 2 (class 3) obesity levels, p=0.004; p=0.023). The predominance of *Candida* sp. in the saliva of women with diabetes/prediabetes can be explained by the fact that diabetes is a metabolic disorder that increases the likelihood of a fungal infection, especially that associated with *Candida* sp. pathogens, due to an immunosuppressive effect of the disease on the patient (*38*). Also, it has been proven that the fungi belonging to *Ascomycota* phylum produce biologically active secondary metabolites that impact diabetes and cardiovascular disorders (*39*). Because of the too general hypothesis, we had many *post hoc* observations like the present one, which had the potential impact on the quality of the work.

In general, in the saliva of obese women with concentrations of 25(OH)D in blood ≥50 nmol/L significantly more species of *Actinobacteria* were found than in those with lower values (p=0.029). In the study of Gominak (*31*), they hypothesized that the combination of serum 25(OH)D and vitamin B creates an environment that favours increase of the number of *Actinobacteria*, *Firmicutes*, *Bacteroidetes* and *Proteobacteria* (normal human microbiome). The same four bacterial species are found in every human with a healthy gut all over the world, so they are called the healthy foursome. The oral vitamin D supplementation in healthy volunteers has decreased the relative amount of pathogenic *Escherichia* spp., *Shigella* spp., *Helicobacter* spp. and *Pseudomonas* spp. (*40*). Long-term vitamin D deficiency may produce changes in the microbiota composition that promoted body mass gain and permanently changed environmental conditions that no longer favour the normal human microbiota. On the other hand, lower serum 25(OH)D values change the microbiota composition, reducing vitamin B production in the gut. With the decrease in *Actinobacteria* in the gut, the vitamin B_12_ blood level was also lower. Therefore, vitamin B_12_, as it exists in nature, may be produced by *Actinobacteria* (*41*).

Female individuals with concentrations of 25(OH)D in blood <50 nmol/L had a statistically significant increase of *Candida* sp. (p=0.022) and *Lactobacillus* sp. in the saliva (p=0.027). Since class 3 obesity was the most prevalent in the group with lower concentration in blood of 25(OH)D (<50 nmol/L), it can be concluded that the presence of *Candida* sp. and *Lactobacillus* sp. in the mouth of obese women is associated with vitamin D deficiency and morbid obesity. In our previous study (*20*), significantly more *Lactobacillus* sp. were observed in the saliva of obese women than in controls with normal body mass, which was explained by their increased consumption of snacks and food rich in sugar. Vitamin D has a signalling role in modulating the host immune system. Vitamin D/vitamin D receptor (VDR) signalling is a major contributor to the gut microbiome at the genetic, environmental and immune levels (*42*). *VDR* gene is the first gene identified as a factor that shapes the gut microbiome at the genetic level. *VDR* knock-out decreased the level of *Lactobacillus* and increased the level of *Clostridium* and *Bacteroides* in mice faeces (*43*). Furthermore, one of the roles of vitamin D in the human immune response is to induce an antimicrobial peptide called cathelicidin LL-37. Cathelicidin is a critical component for the body's ability to fight infections, such as candidiasis (*44*). This could be the reason for the increased oral amount of *Candida* sp. in the saliva of obese women with a vitamin D deficiency. In this study, the presence of *Candida* sp. was also positively associated with blood glucose concentrations (diabetes/prediabetes). Our results confirm those by Man *et al.* (*45*), who also showed that higher glucose concentration is directly related to *Candida albicans* growth, which may be associated with fungal infections that occur in non-controlled diabetic patients.

The mechanism of microbiota composition by which bacterial and fungal metabolisms impact the concentration of vitamin D in obese women is less known. Vitamin D can directly control the immune system by VDR signalling. On the other hand, some microbes like *Lactobacillus reuteri* increased 25(OH)D concentrations (*46*). Supplementation with *Lactobacillus rhamnosus* and *Lactobacillus plantarum* enhances the expression of VDR in intestinal epithelial cells (*47*). Khosravi *et al*. (*48*) showed that the increase of 25(OH)D in the blood had a positive effect on body mass loss, which is partly attributed to the anti-inflammatory effects.

Our study is subject to several limitations. We performed a preliminary study with only the female population involved. Defined selection reasons are given in the Methods, paragraph Subjects and study design. Briefly, differences in diet, age and hormonal factors may affect the development of obesity. In further research we plan to conduct a study on the male population. Since the initial hypothesis that changing trend of oral microbiota composition depends on micronutrient concentrations and/or presence of various obesity-related diseases in obese women in Croatia was not precise enough, manipulating a large amount of data generated a number of *post hoc* observations that contributed to the overall quality of the work. Therefore, this study can be defined in part as exploratory and hypothesis-generating.

## CONCLUSIONS

There is no relationship between micronutrient deficiency and imbalanced oral microbiota. On the other hand, we showed positive correlations among the incidence of hypertension or diabetes/prediabetes, BMI values and pathogenic bacteria in the oral cavity of obese women in Croatia. This study highlights the potential influence of oral microbiota (with a predominance of probiotic bacteria) on BMI reduction and stabilization of obesity-related diseases in obese women in Croatia.
